# Review of "Ants, Bikes & Clocks: Problem Solving for Undergraduates", by William Briggs

**DOI:** 10.1186/1475-925X-4-45

**Published:** 2005-07-24

**Authors:** Eduardo Abreu

**Affiliations:** 1 Childrens' Hospital of Boston, Department of Orthopaedic Surgery, Enders 1022, 300 Longwood Street, Boston MA 02115, USA

Probably, most of the students who have taken (or will take) undergraduate courses based on the so-called exact sciences have faced (or will face at some point) the terror of being unable to solve some of the proposed problems or exercises, despite having a good theoretical knowledge of the topic. Basically, you may know the equations, you may understand the mathematical passages, you may have a grasp on the physical meaning of the equations and yet sometimes you are unable to get past the problem's enunciate! Unfortunately, this inability to solve problems often leads to frustration. And this feeling of helplessness starts a vicious circle, as the incapability of solving some problems results in diminished motivation to study and, as a consequence, more difficulties in solving future problems. Surely, problem solving is an important and complex aspect of our education; however it is frequently overlooked by teachers. For this reason, a book aimed at the development of problem solving skills should be welcomed by students and teachers.

More than 50 years ago, G. Polya wrote a book on problem solving strategies which became very popular, "How to Solve It" [1]. Brigg's book is not intended to replace, but rather to complement Polya's classic book. For example, not only Polya's principles of analytical problem solving are cited and discussed in Brigg's book, but they are also adapted to computational problems. In fact, the interplay between the analytical and computational approaches to problem solving is very useful since computers became part of our daily lives.

One of the main qualities of "Ants, Bikes & Clocks: Problem Solving for Undergraduates" is that it is a pleasant book to read. It is well written and the understanding of the text is facilitated by numerous tables, graphs, charts and figures. This readability is especially important considering the main target of the book, undergraduate students. But everyone would certainly enjoy browsing the chapters, reading the small notes on the sides of the pages, going through the various and inventive examples, etc. I did it! Another interesting aspect of Brigg's book is how the exercises are organized. At the end of the chapters, each exercise is followed by hints and respective answer. In addition, full solutions for all exercises can be found at the end of the book. Although the author cautions against checking the full solutions before trying to solve the problems, it is certainly good to know that the full solutions are within reach.

In summary, this is an excellent and opportune book about problem solving, not only for undergraduates, but for everyone who is interested in problem solving strategies.
